# Supplementary material for: Insights into phosphoethanolamine cellulose synthesis and secretion across the Gram-negative cell envelope
Source: Nat Commun. 2024 Sep 6;15:7798. doi: 10.1038/s41467-024-51838-0 (PMC11379886; doi:10.1038/s41467-024-51838-0)
Supplement: Supplementary file 1 — Supplementary Information [file 41467_2024_51838_MOESM1_ESM.pdf]

Insights into phosphoethanolamine cellulose synthesis and secretion across the Gram-negative cell envelope

Preeti Verma[1], Ruoya Ho[1], Schuyler A. Chambers[2], Lynette Cegelski*[2], Jochen Zimmer*[1,3]

Affiliations

1 Department of Molecular Physiology and Biological Physics, University of Virginia School of Medicine, Charlottesville, VA 22903, USA

2 Department of Chemistry, Stanford University, Stanford, CA 94305, United States

3 Howard Hughes Medical Institute

Contact

* Email: cegelski@stanford.edu, jochen_zimmer@virginia.edu

Supplementary Information

Supplementary Figures S1-S13

Table S1 and S2

Supplemental Figures and Captions

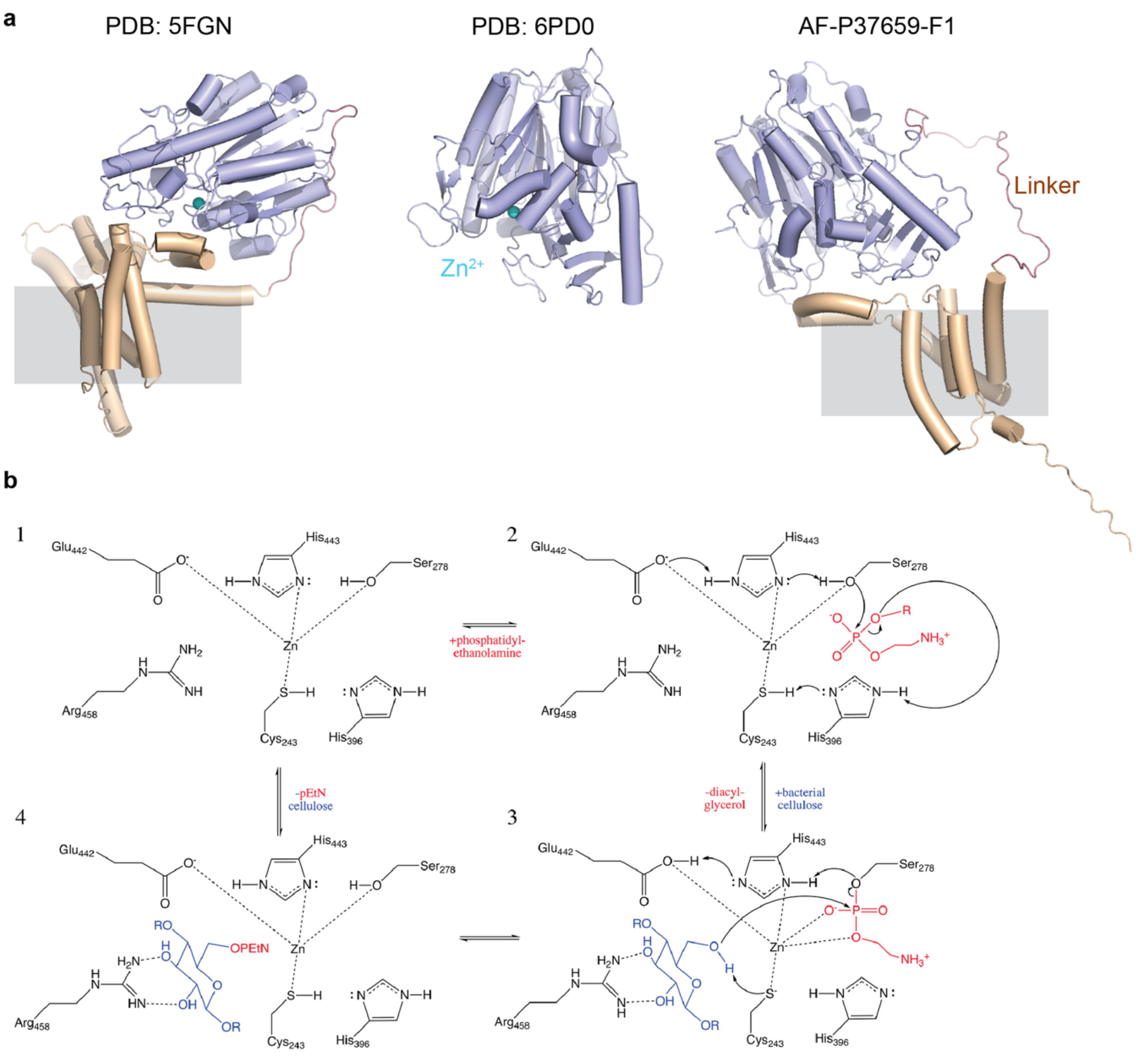

https://creativecommons.org/licenses/by-nc-nd/4.0/

Fig. S1: BcsG is a phosphoethanolamine transferase. a, Comparison of the crystal structures of EptA (PDB: 5FGN) and the catalytic domain of BcsG (PDB: 6PD0) with the AlphaFold-2 predicted full-length structure of BcsG. **b**, Proposed BcsG reaction mechanism as presented in reference [25].

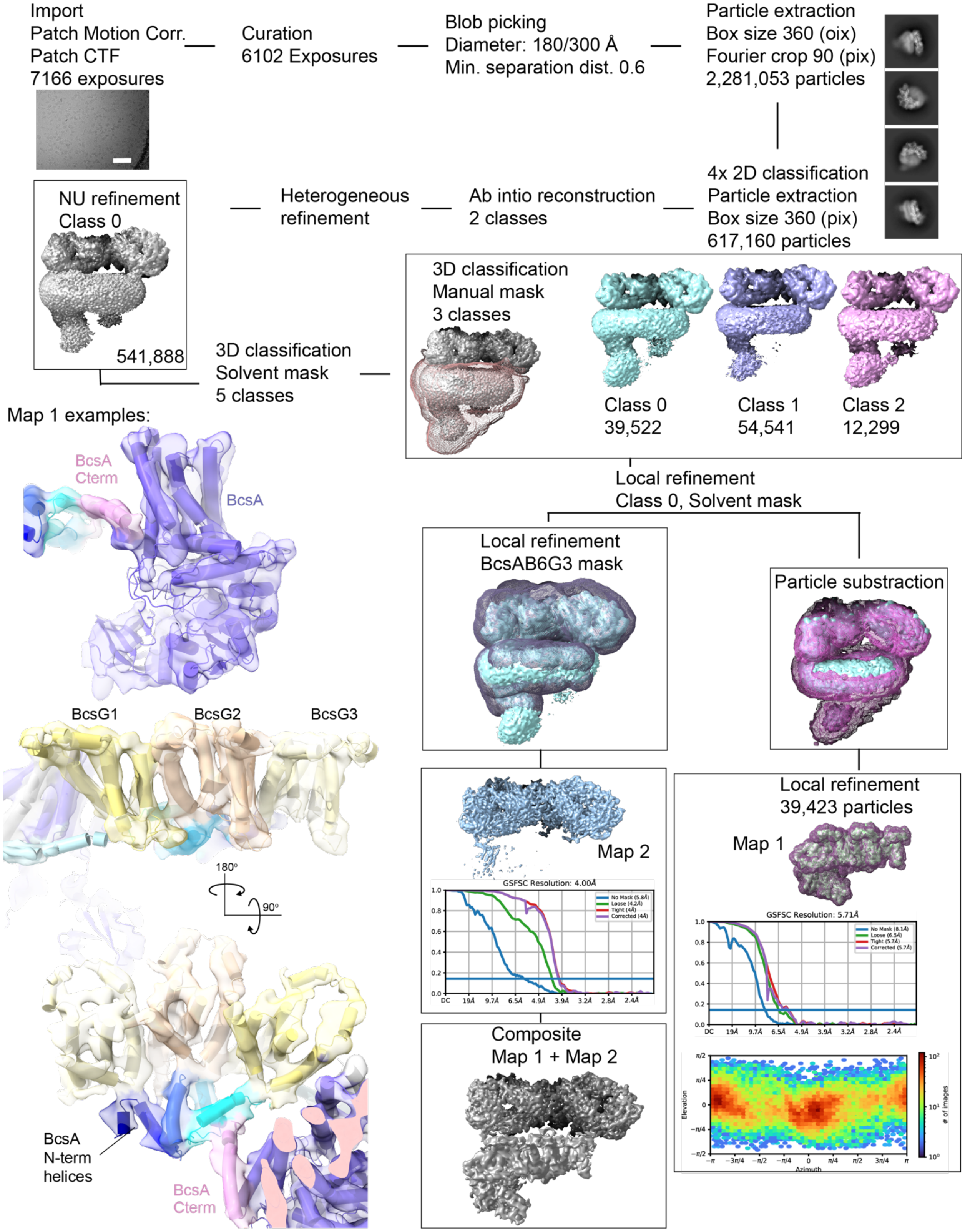

Fig. S2: Workflow of cryo-EM data processing of the *Ec* BcsAB6G3 complex. Revised processing of entry EMD-23267. Scale bar on the micrograph corresponds to 100 nm. The map colored to local resolution is provided in Supplementary Figure S3.

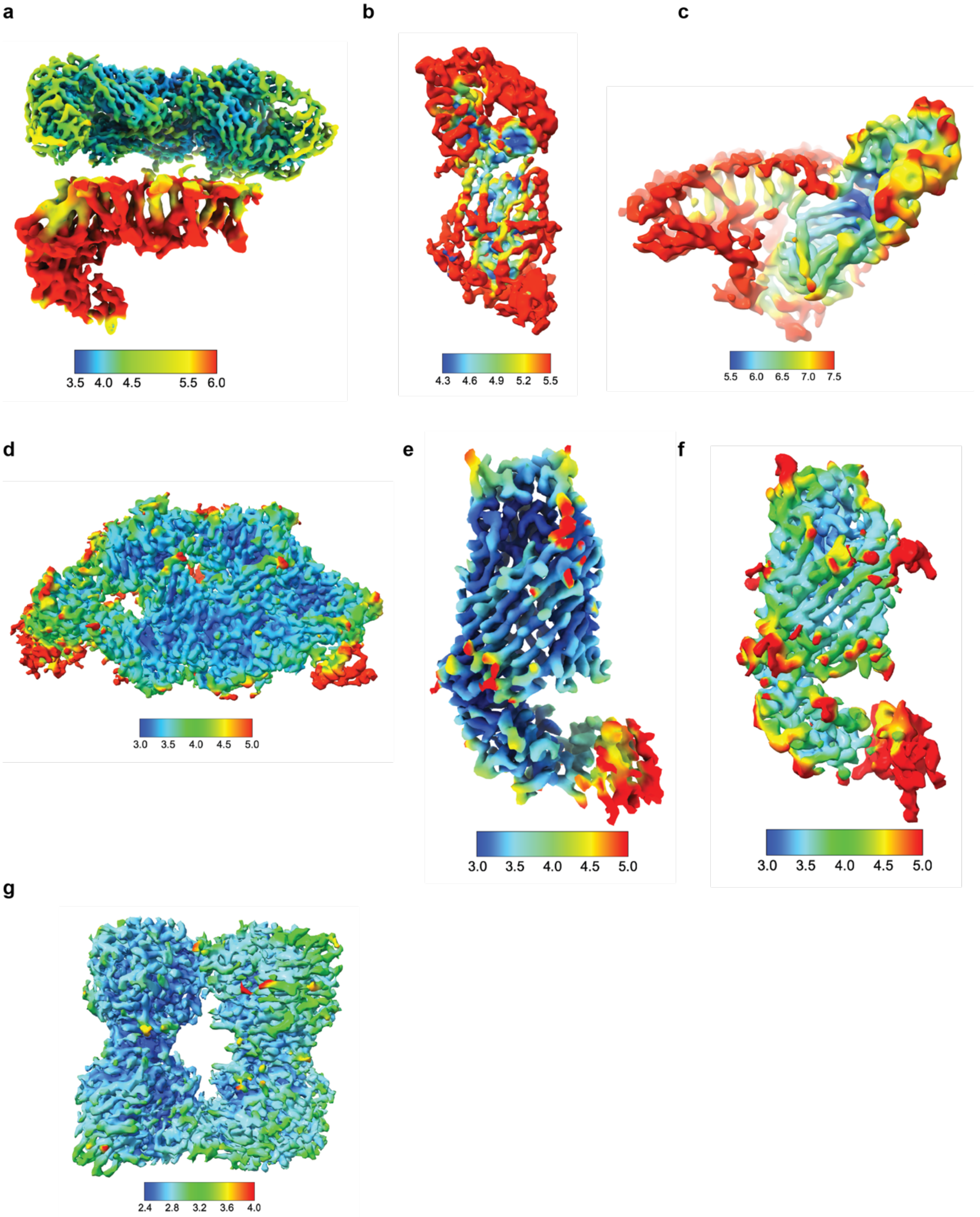

Fig. S3 Local resolution of the final map. a, *Ec* BcsAB6G3 complex. **b-c,** Chimeric BcsAB-BcsG complex. **d,** BcsB-BcsC fusion *Ec* complex **e,** BcsC apo. **f,** Bcs Cellotetraose bound. **g,** BcsZ. Resolution estimates are in Å.

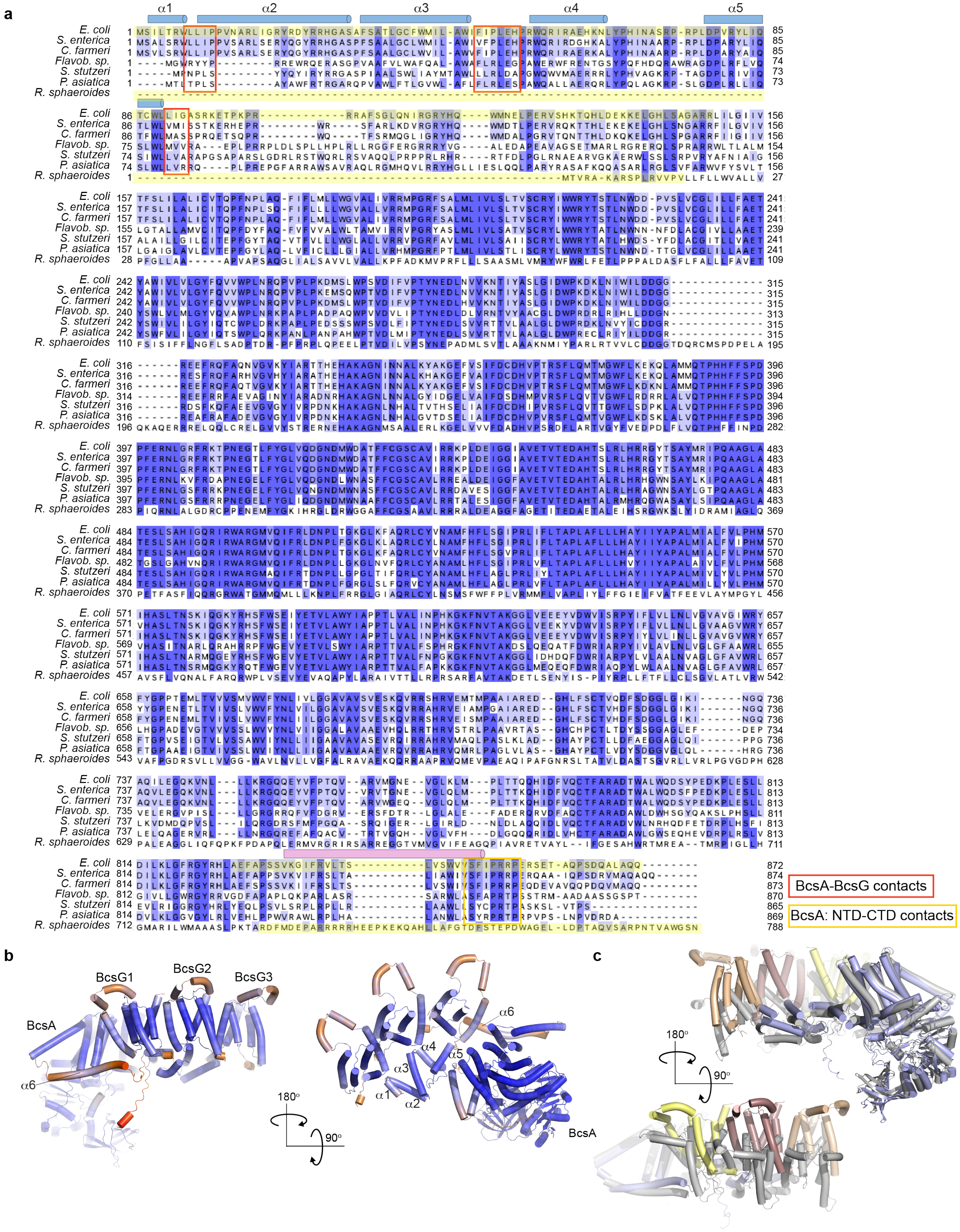
a
α1
α2
α3
α4
α5
E. coli
S. enterica
C. farmeri
Flavob. sp.
S. stutzeri
P. asiatica
R. sphaeroides
BcsA-BcsG contacts
BcsA: NTD-CTD contacts
b
BcsG1
BcsG2
BcsG3
BcsA
α6
α6
α4
α5
α3
α1
α2
180°
90°
BcsA
c
180°
90°

Fig. S4: Sequence alignment of BcsA and its interaction with BcsG. a, Sequences were aligned in MUSCLE[43] and displayed in Jalview[44]. N- and C-terminal helical elements are shown as cylinders in blue and pink, respectively. Both the regions in *R. sphaeroides* and the corresponding regions from *E. coli* that replace them are highlighted in yellow boxes. **b**, AlphaFold2-predicted complex of BcsA with three copies of BcsG's TM segment, colored based on pLDDT score from blue to red for high to low confidence scores. **c**, Overlay of the generated AlphaFold2 predicted complex of the *Ec* BcsA-BcsG3 complex (colored blue for BcsA and yellow, violet and wheat for the BcsG subunits) and the model obtained after rigid body docking of the subunits into the experimental cryo EM map (colored gray).

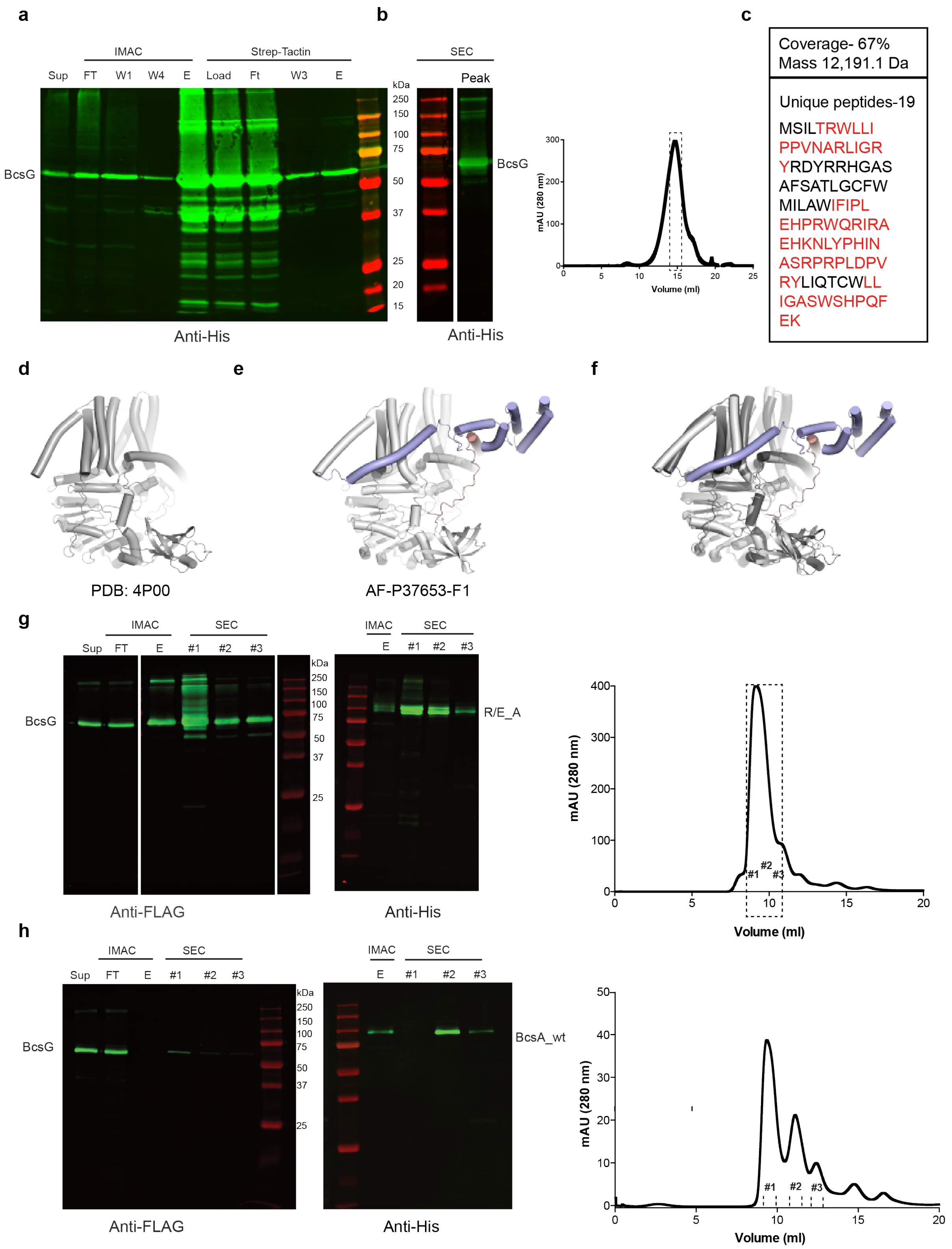

Fig. S5: Interactions of BcsA and BcsG. a, Anti-His Western blot of samples obtained from a tandem purification using IMAC and Strep-Tactin affinity matrices. BcsG is His-tagged and BcsA's

NTD is Strep-tagged. Sup: Supernatant, FT: flow through, W: Wash step, E: Elution. **b**, Anti-His Western blot of the peak fraction after size exclusion chromatography (SEC). Right panel: Superose 6 increase SEC profile of the co-purified BcsA NTD-BcsG complex. **c**, Peptide coverage (red) of BcsA's NTD after mass spectrometry fingerprinting of a gel slice excised from an acrylamide gel at the expected molecular weight. **d** and **e**, Comparison of *Rs* (PDB: 4P00) and Ec BcsA (AF_P37653-F1). **f**, Superposition of *Rs* BcsA (PDB: 4P00) with *Ec* BcsA (AF_P37653-F1) by secondary structure matching. Regions absent in *Rs* BcsA are shown in blue (*Ec* BcsA NTD) and red (*Ec* BcsA C- terminus). **g**, Western blot detecting FLAG-tagged BcsG and the His-tagged chimeric BcsA (R/E_A) after IMAC and SEC for the purified chimeric BcsAB-BcsG complex. Right panel: Supedex200 increase SEC profile**.** Sup: Supernatant, FT: flow through, E: Elution, #1-3: SEC fractions. **h**, Western blot detecting FLAG-tagged BcsG and His-tagged BcsA, respectively, of samples obtained after IMAC and SEC for the wild type BcsAB complex co-expressed with *Ec* BcsG and BcsF. Right panel: Supedex200 increase SEC profile. Solubilized membranes (Sup) have been loaded as reference for BcsG expression in the Anti-FLAG blot. Source data are provided as a Source Data file.

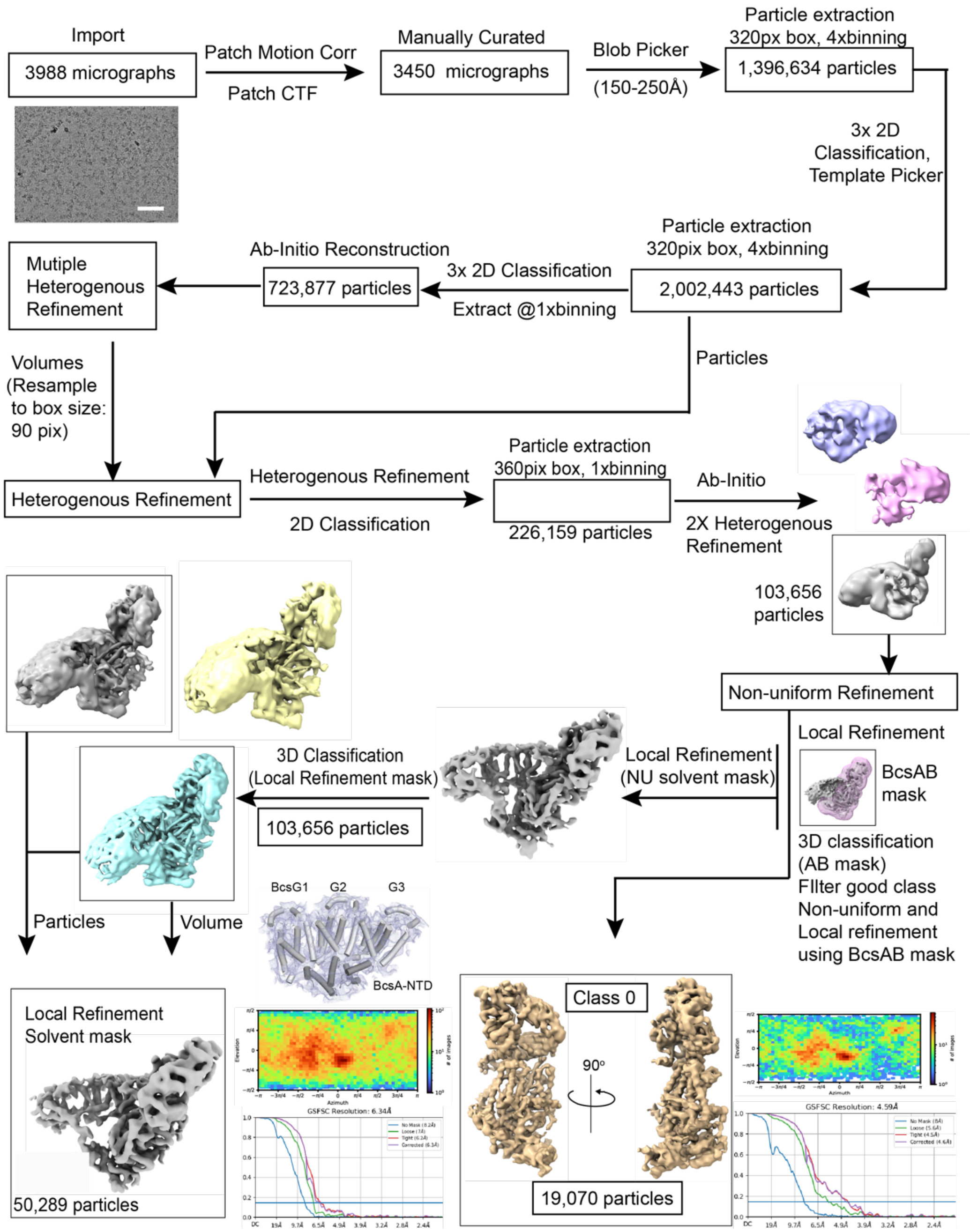

Fig. S6: Workflow of cryo-EM data processing of the chimeric BcsAB-BcsG complex. Scale bar on the micrograph corresponds to 100 nm. Map quality of BcsA-NTD and BcsG3 are shown with maps contoured at σ=6 (BcsA NTD helices are colored dark grey and BcsG copies are colored light grey). Maps colored to local resolution are provided in Supplementary Figure S3.

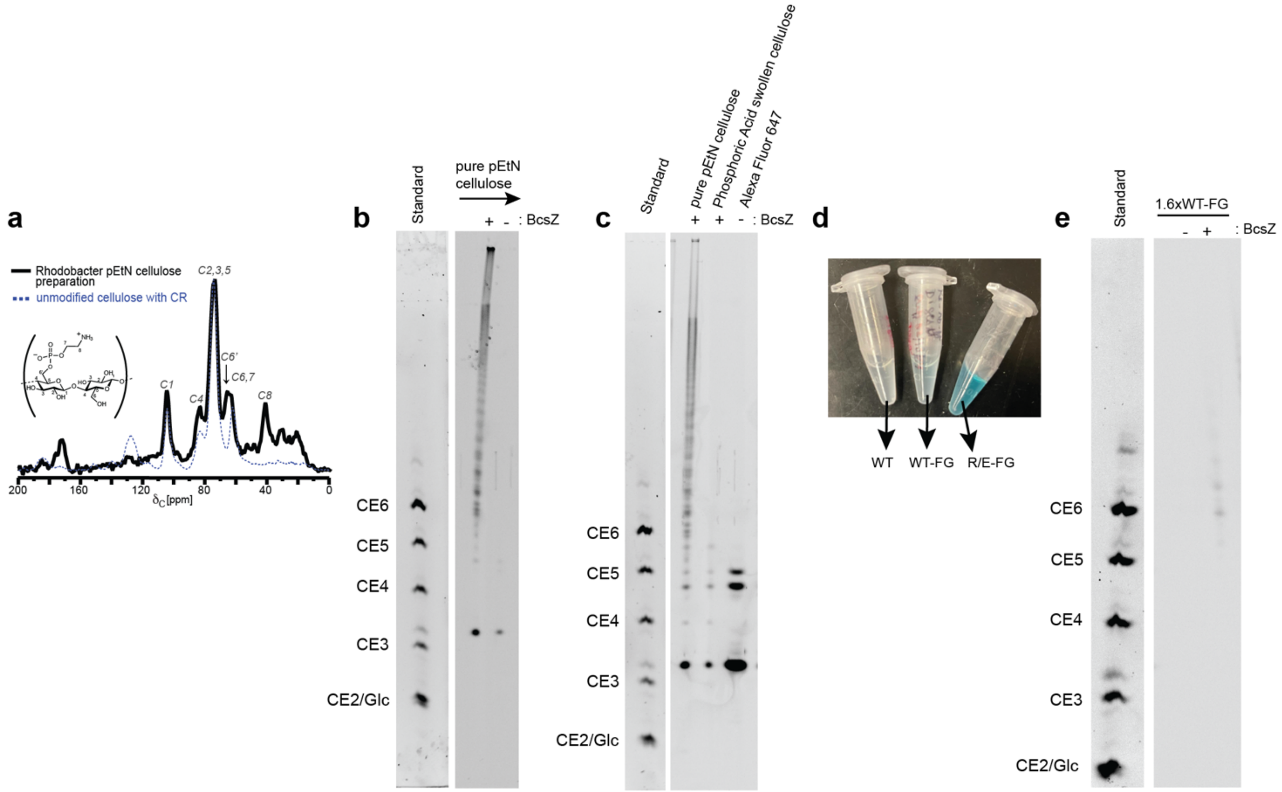

Fig. S7: Detection of pEtN-cellulose by solid-state NMR and polysaccharide carbohydrate gel electrophoresis (PACE). **a**, Solid-state NMR spectrum of pEtN cellulose synthesized by the chimeric BcsAB-BcsG complex *in vivo* (R/E-FG; black line), overlaid with the reference spectrum of unmodified cellulose purified with CR (dashed blue line). Peaks labeled C1-8 correspond to the chemical shifts expected for the indicated carbon atoms (see inset). The inset represents the chemical structure of the glucosyl and pEtN glucosyl units in pEtN modified cellulose with carbon atoms numbered. **b**, Control labeling of purified pEtN-cellulose produced by *Ec* with Alexa Fluor 647 NHS ester followed by cellulase digestion (BcsZ). **c**, Similar as for panel (b) but including digestion of phosphoric acid swollen cellulose and an Alexa Fluor 647 NHS ester-only sample. **d**, Supernatants containing the released soluble cello-oligosaccharides after cellulase (BcsZ) digestion as described in Methods. **e**, As for panels b and c, PACE analysis of cellulose produced by IMVs containing the WT BcsAB complex co-expressed with BcsG and BcsF. 1.6-times more IMVs were used for cellulose synthesis reaction as compared to Fig. 2h. Standard: Mixture of ANTS labeled cello-oligosaccharides (DP 2 to 6) and glucose. PACE experiments have been performed two times with similar results. Source data are provided as a Source Data file.

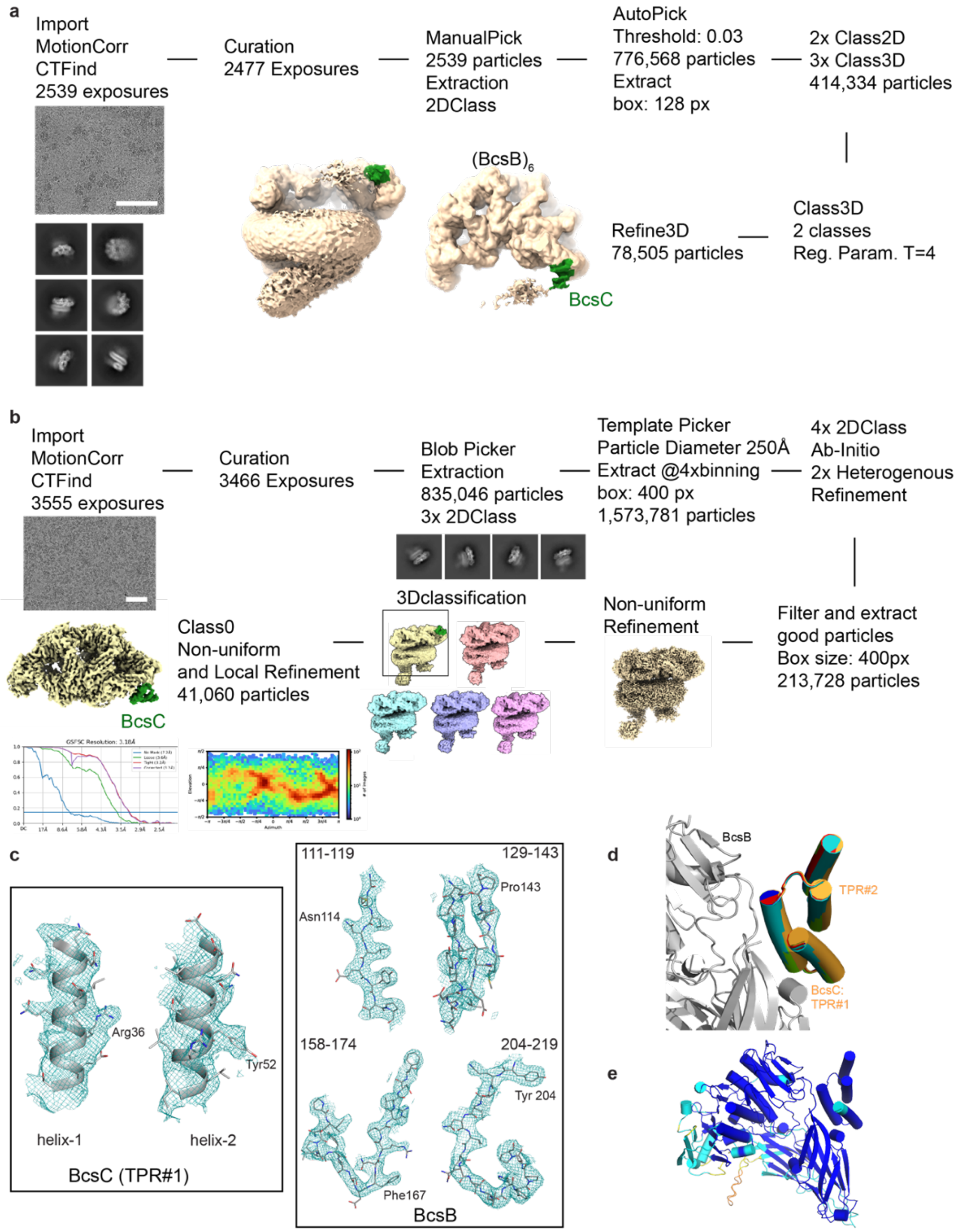

Fig. S8: Interaction of BcsB and BcsC. **a**, RELION Workflow of cryo-EM data processing of the Bcs complex in the presence of BcsC's periplasmic domain. **b**, CryoSPARC workflow of cryo-EM data processing of the Bcs complex containing BcsC's N-terminal two TPRs fused to BcsB (BcsB-BcsC fusion *Ec* complex). The map colored to local resolution is provided in Supplementary Figure S3. c, Examples of the map quality shown for TPR#1 as well region of BcsB that interacts with TPR#1, contoured at σ=5 for BcsC and σ=7 for BcsB. **d**, Five predicted AlphaFold2 models of BcsB in complex with BcsC's N-terminal two TPRs. BcsB for one of the models is shown as grey cartoon and BcsC TPR helices represented as cylinders are shown in different colors for models 1-5. **e**, As for panel d, but colored based on pLDDT score (blue to red for high to low confidence). Scale bar on the micrograph corresponds to 100 nm.

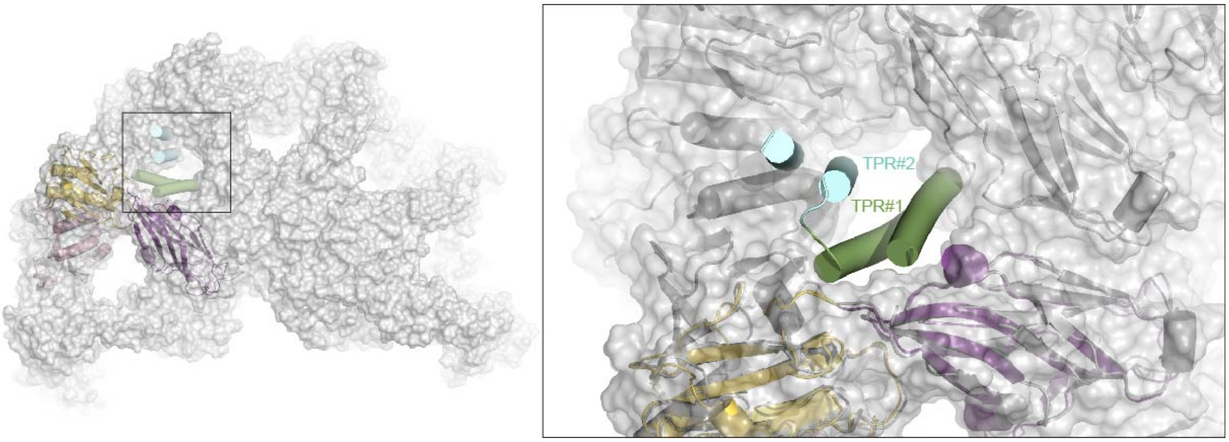

Fig. S9 Steric constraints of BcsB and BcsC interaction. The terminal sixth copy of BcsB in complex with BcsC was aligned with the second protomer of the BcsB semicircle to highlight steric clashes.

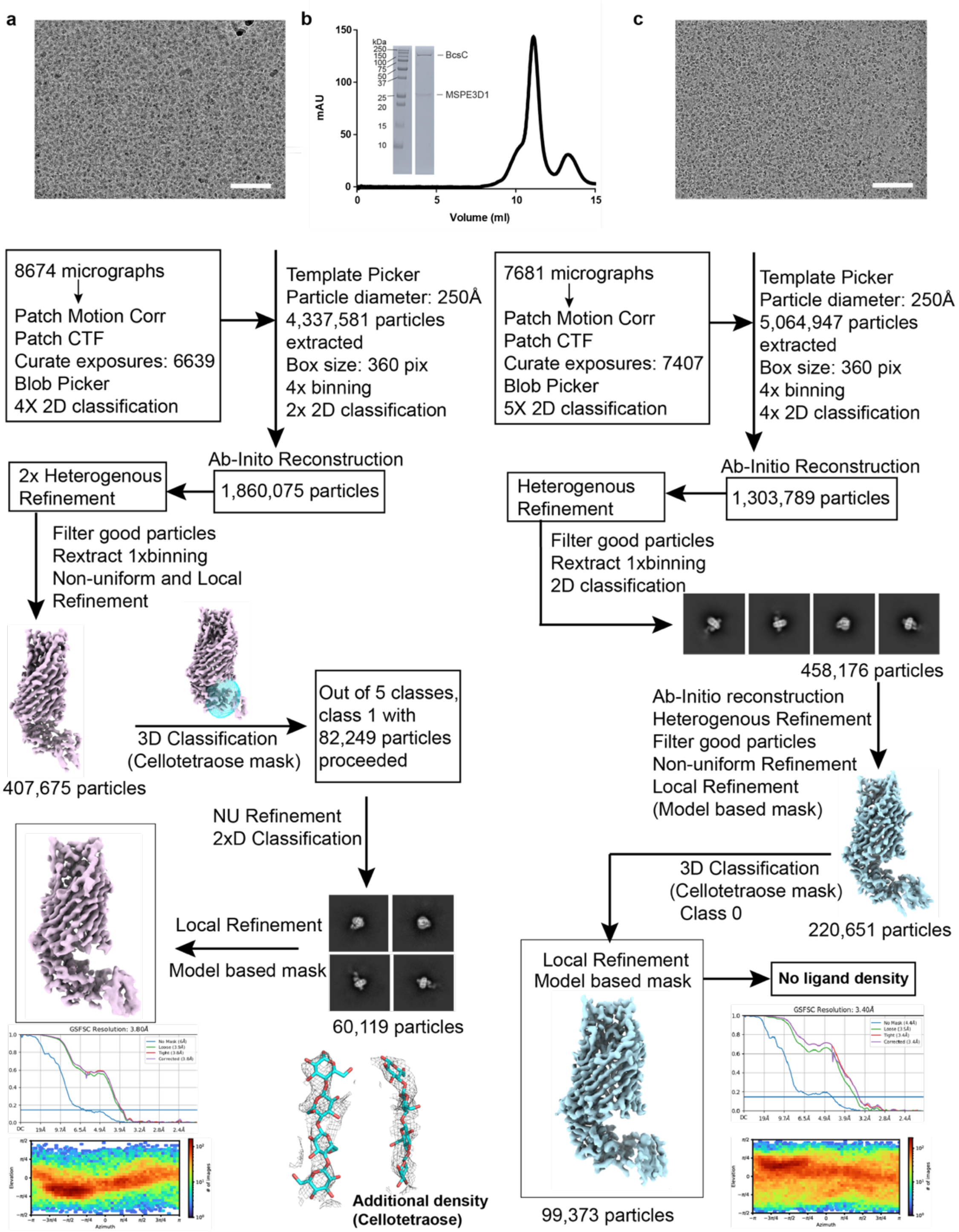

Fig. S10 Workflow of cryo-EM data processing of BcsC with and without cellotetraose. a, Workflow in the presence of cellotetraose with ligand density contoured at 11 σ and shown as black mesh. **b**, Gel filtration chromatogram of nanodisc-reconstituted BcsC. Inset: Coomassie-stained SDS-PAGE of the peak fraction. **c**, Workflow in the absence of cellotetraose. Scale bar on the micrograph corresponds to 100 nm. The maps colored to local resolution are provided in Supplementary Figure S3. Source data are provided as a Source Data file.

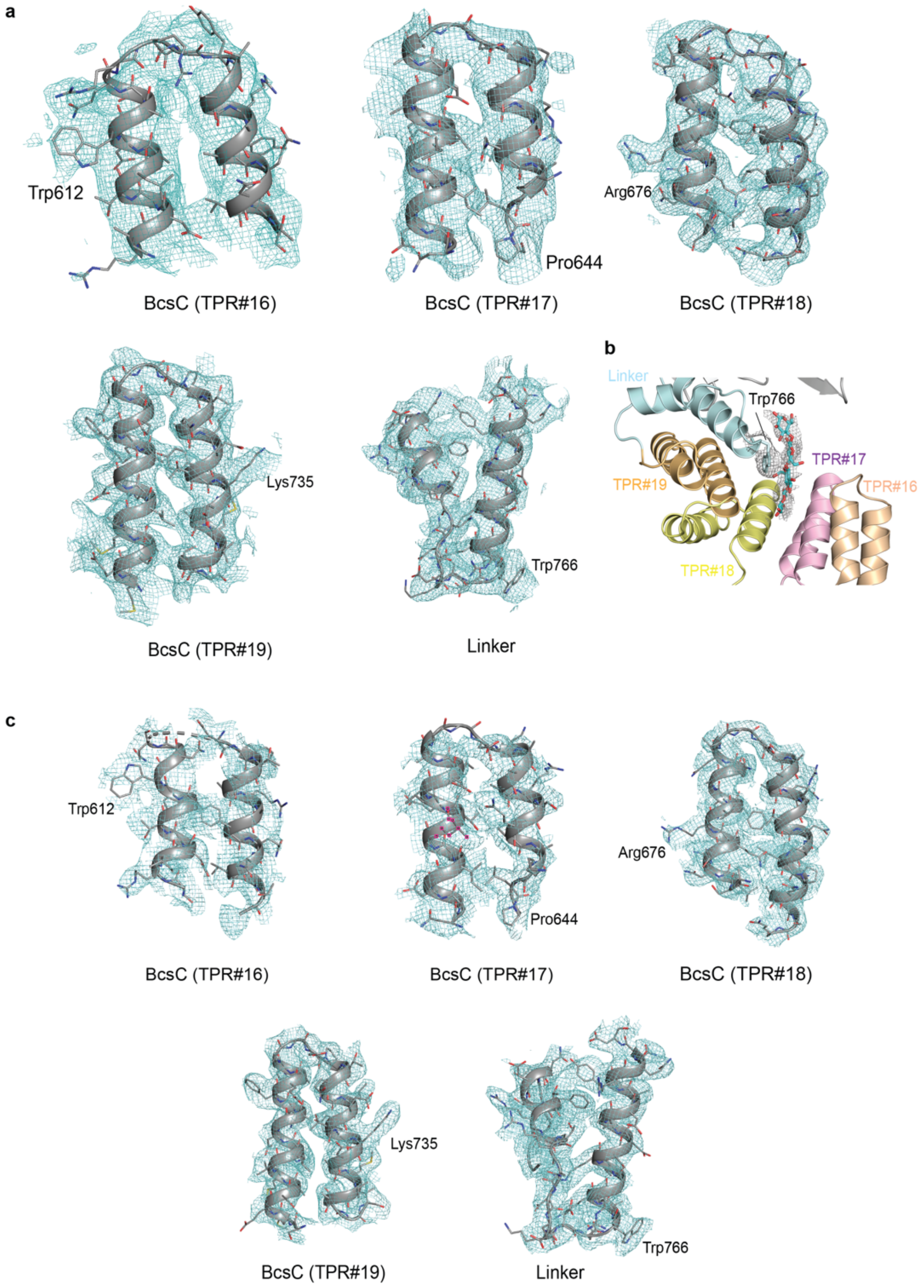

Fig. S11 Examples of map quality of TPR#16-19 and the linker region in BcsC with and without cellotetraose (contoured at σ=11). a, BcsC bound to cellotetraose **b**, Cryo-EM density map Trp766 proximal to cellotetraose. TRP#16, 17, 18, 19 and the linker region shown as cartoons are colored in wheat, pink, yellow, orange and cyan respectively and cellotetraose is shown as sticks colored cyan and red. **c**, BcsC without cellotetraose.

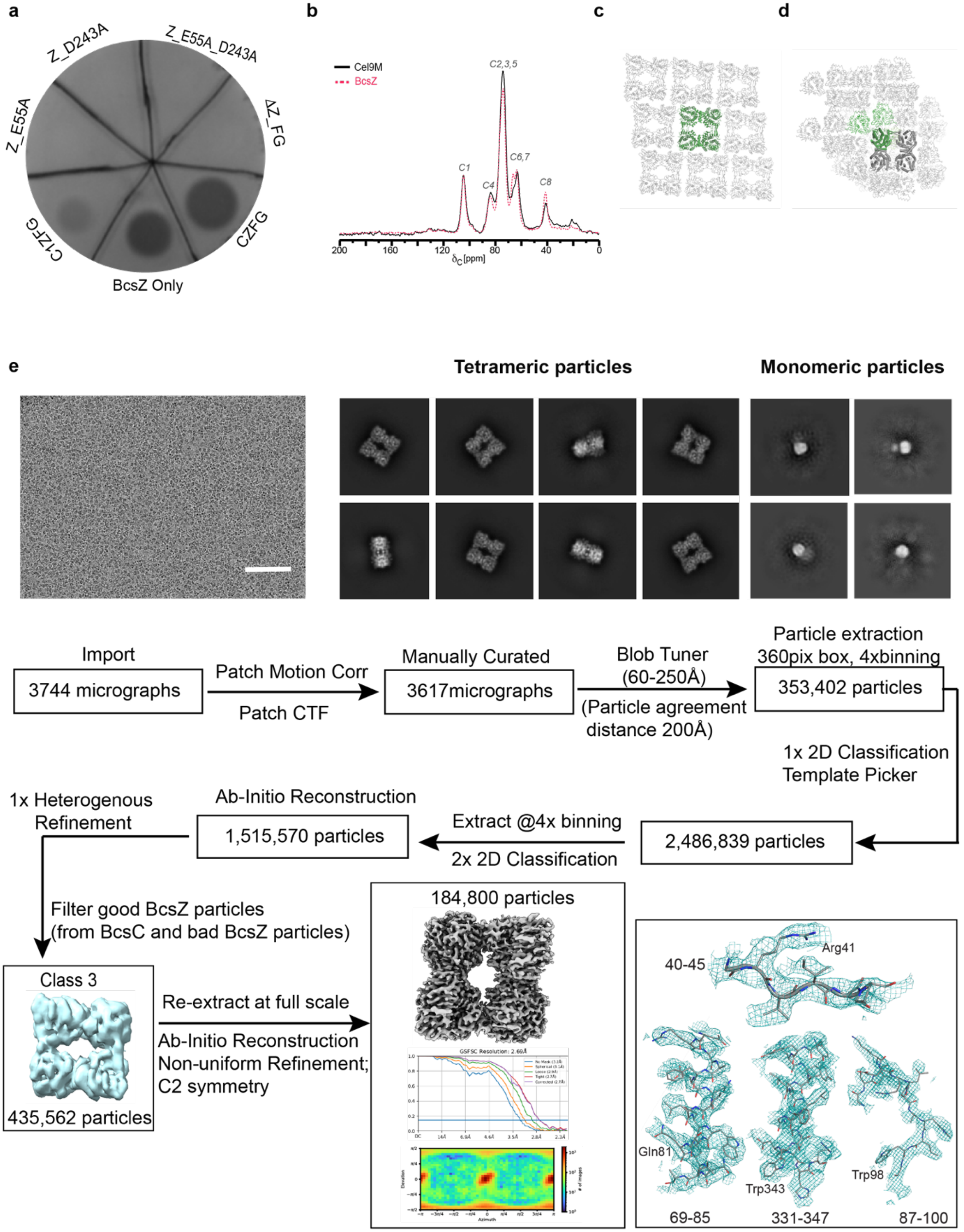
a
Z_D243A
Z_E55A_D243A
ΔZ_FG
Z_E55A
CZFG
C1ZFG
BcsZ Only
b
Cel9M
BcsZ
C2,3,5
C6,7
C1
C4
C8
200
160
120
80
40
0
δC [ppm]
c
d
e
Tetrameric particles
Monomeric particles
Import
3744 micrographs
Patch Motion Corr
Patch CTF
Manually Curated
3617micrographs
Blob Tuner
(60-250Å)
(Particle agreement
distance 200Å)
Particle extraction
360pix box, 4xbinning
353,402 particles
1x 2D Classification
Template Picker
2,486,839 particles
Extract @4x binning
2x 2D Classification
Ab-Initio Reconstruction
1,515,570 particles
1x Heterogenous
Refinement
Filter good BcsZ particles
(from BcsC and bad BcsZ particles)
Class 3
435,562 particles
Re-extract at full scale
Ab-Initio Reconstruction
Non-uniform Refinement;
C2 symmetry
184,800 particles
40-45
Arg41
Gln81
Trp343
Trp98
69-85
331-347
87-100

Fig. S12 Function and structure of BcsZ. **a**, Cellulase activity of overnight *Ec* cultures expressing the indicated BcsZ variants along with the full length BcsC, BcsF and BcsG subunits. ∆BcsZ: no BcsZ. BcsZ Only- indicates *Ec* cells expressing only WT BcsZ, CZFG indicates cells expressing the full-length BcsC, BcsZ, BcsF and BcsG subunits. C1ZFG: As for CZFG but expressing a BcsC construct lacking the TPRs. Cells were spotted on carboxymethylcellulose-containing agar plates, incubated at 37 °C for 48 hours and stained with CR as detailed in Methods. Cellulase plate assays were repeated at least three times with similar results. **b** Natural abundance ^{13}C CPMAS NMR spectra of pEtN cellulose isolated from the C43 cells expressing the *Ec* Bcs TMC complex (BcsZ; dashed red line) and pEtN cellulose produced *in vivo* by C43 cells expressing *Ec* Bcs complex along with Cel9M in place of BcsZ (Cel9M; solid black line). **c** and **d**, Crystal packing of BcsZ in space group P21 and P1, PDB entries 3QXQ and 3QXF. **e**, Workflow of cryo-EM data processing of BcsZ including a representative micrograph and 2D class averages**.** Scale bar on the micrograph: 100 nm. Examples of EM map quality, contoured at σ=7. The map colored to local resolution is provided in Supplementary Figure S3. Source data are provided as a Source Data file.

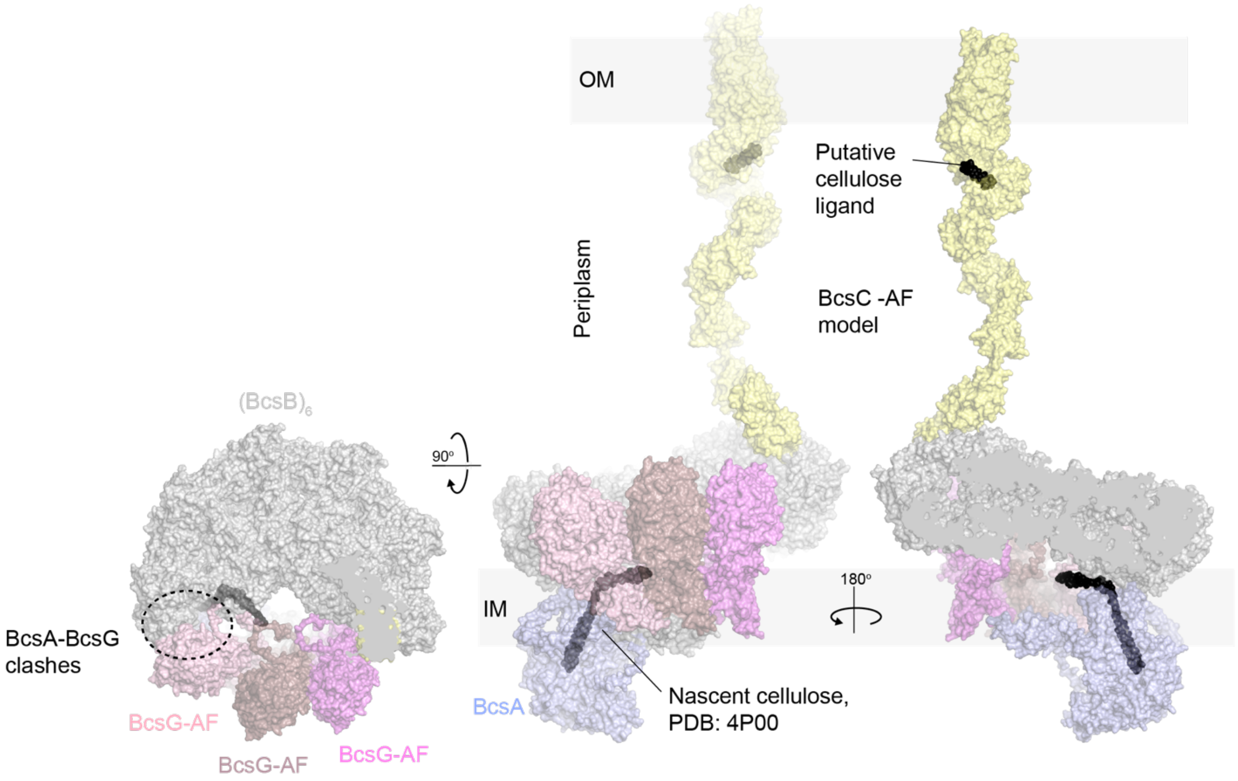

Fig. S13 Pseudo-atomic model of the *Ec* Bcs complex with a focus on pEtN modification and secretion. The AlphaFold2 predicted full-length structures of *E. coli* BcsG (AF-P37659-F1) and BcsC (AF-P37650-F1) were docked into the experimentally observed densities of their TM segments and N-terminal TPR, respectively. The periplasmic domain of BcsC was manually adjusted to establish a 'straight' conformation of its TPR solenoid. Clashes between the first BcsG subunit (colored light pink) and the first BcsB subunit associated with BcsA are evident (dashed oval), indicating that the catalytic domain of three copies of BcsG cannot be accommodated in this fashion and will require re-orientation. The nascent cellulose polymer observed in the *Rhodobacter sphaeroides* BcsA structure was added to the *E. coli* BcsA model after superimposition by secondary structure matching in Coot (shown as thick black sticks). Cellotetraose, bound to BcsC as observed in the cryo-EM maps, is also shown in sticks and labeled as 'putative cellulose ligand'.

Table S1: Cryo-EM data statistics and refinement.

	Ec BcsAB6G3 (EMD-44359) (PDB 9B8V)	Chimeric-BcsAB-BcsG complex	*Ec* Bcs complex with BcsC (#TPR1-18)	BcsB-BcsC fusion *Ec* complex (EMD-44346) (PDB 9B8I)	BcsC Apo (EMD-44336) (PDB 9B8A)	BcsC Cellotetraose bound (EMD-44345) (PDB 9B8H)	BcsZ (EMD-44334) (PDB 9B87)
**Data collection and processing**							
Magnification	81,000x	81,000x	81,000x	81,000x	81,000x	81,000x	81,000x
Voltage (kV)	300	300	300	300	300	300	300
Electron exposure (e–/Å^2)	51	50	50	50	50	50	50
Defocus range (μm)	-2.5 to -1	-2 to -1	-1.8 to -0.8	-2 to -1	-2.2 to -1.2	-2.4 to -1.2	-2.2 to -1.0
Pixel size (Å)	1.08	1.08	1.08	1.08	1.08	1.08	1.08
Symmetry imposed	C1	C1	C1	C1	C1	C1	C2
Initial particle images (no.)	2,281,053	2,002,443	776,568	1,573,781	5,064,947	4,337,581	2,111,486
Final particle images (no.)	39,522	50,289	78,505	41,060	99,373	60,119	184,800
Map resolution (Å)	4	6.3	12	3.2	3.4	3.8	2.7
FSC threshold	0.143	0.143	0.143	0.143	0.143	0.143	0.143
Map resolution range (Å)	5.3-100	2.2-98.8		2.35-51.6		3.1-56.3	2.4-39
**Refinement**							
Initial model used (PDB code)	AlphaFold2 and 7L2Z	AlphaFold2	AlphaFold2	AlphaFold2	AlphaFold2	AlphaFold2	3QXQ
Model resolution (Å)	5.7			3.2	3.4	3.8	2.60
FSC threshold=0.143							
Map sharpening *B* factor (Å^2)	347			-58.7	-65.9	-78.3	-80.1
Model composition							
Non-hydrogen atoms	70369			5610	4320	4630	10912
Protein residues	5179			719	554	587	1352
Ligands	0			0	0	CE4: 1	0
B factors (Å^2)	Rigid body						
Protein	docked			119.68	63.78	84.55	27.46
Ligand	only			-		102.63	-
R.m.s. deviations							
Bond lengths (Å)				0.005	0.005	0.006	0.009
Bond angles (°)				1.000	0.968	0.983	1.368
Validation							
MolProbity score				1.37	1.11	1.61	1.24
Clashscore				4.20	3.22	5.35	3.35
Poor rotamers (%)				0.96	0.89	0.42	0.62
Ramachandran plot							
Favored (%)				97.05	98.18	95.38	97.40
Allowed (%)				2.95	1.82	4.62	2.60
Disallowed (%)				0	0	0	0

Table S2: Primers used in this study.

S.No.	Primer Name	Sequence
1.	EcBcsG_Fw	ATAATGAGCCATGGGCACTCAATTTACGCAAAATACCGCCATG
2.	EcBcsG_His_Rv	CAGCGATCTCGAGTCAGTGATGGTGATGGTGATGGTGATGGTGAT GGTGATGCTGCGGGTAAGGCACCCAGTCGC
3.	EcBcsA_Fw	ATAATGAGCCATGGGTAGTATCCTGACCCGGTGGTTGCTTATC
4.	EcBcsA_Strep_Rv	CAGCGATCTCGAGTTATTTTTCAAACTGCGGATGGCTCCA CGATGCACCGATCAATAACCAGC
5.	AdrA_Fw	ATAATGAGCCATGGATGTTCCCAAAAATAATGAATGATGAAAACTT TTTC
6.	AdrA_Rv	TTTTTCAAACTGCGGATGGCTCCACTAAGCTGGTACCTCAGGCCGC CACTTCGGTGCGGTTACGTCCG
7.	BcsC periplasmic_Fw	ATAATGAGCCATGGCACCAACCGCTCAGCAACAGTTGC
8.	BcsC periplasmic_Rv	CTAAGCTCTCGAGTCAATGATGGTGGTGATGGTGATGGTGATGGT GTTCCATCGACGGTGGCTGAG
9.	BcsZ_E55_Fw	ACTACTAGCGCGGGCCAGAGCTATGGCATGTTCTCTGCCCTGGCG G
10.	BcsZ_E55_Rv	GCTCTGGCCCGCGCTAGTAGTAATTTTGCGCGCGTCGCTGGGGTC G
11.	BcsZ_D243_Fw	TCCTCCTATGCGGCCATTCGTGTGTACATGTGGGTAGGCATGATGC
12.	BcsZ_D243_Rv	ACGAATGGCCGCATAGGAGGAAATCAATGTTTTTTCGGCTTTTAGC
