# Supplementary material for: Insights into phosphoethanolamine cellulose synthesis and secretion across the Gram-negative cell envelope
Source: Nat Commun. 2024 Sep 6;15:7798. doi: 10.1038/s41467-024-51838-0 (PMC11379886; doi:10.1038/s41467-024-51838-0)
Supplement: Supplementary file 3 — Reporting Summary [file 41467_2024_51838_MOESM3_ESM.pdf]

nature portfolio

Corresponding author(s): Zimmer, Jochen

Last updated by author(s): August 4, 2024

Reporting Summary

Nature Portfolio wishes to improve the reproducibility of the work that we publish. This form provides structure for consistency and transparency in reporting. For further information on Nature Portfolio policies, see our Editorial Policies and the Editorial Policy Checklist.

Statistics

For all statistical analyses, confirm that the following items are present in the figure legend, table legend, main text, or Methods section.

n/a	Confirmed	
☐	☒	The exact sample size (*n*) for each experimental group/condition, given as a discrete number and unit of measurement
☐	☒	A statement on whether measurements were taken from distinct samples or whether the same sample was measured repeatedly
☒	☐	The statistical test(s) used AND whether they are one- or two-sided<br>*Only common tests should be described solely by name; describe more complex techniques in the Methods section.*
☒	☐	A description of all covariates tested
☒	☐	A description of any assumptions or corrections, such as tests of normality and adjustment for multiple comparisons
☐	☒	A full description of the statistical parameters including central tendency (e.g. means) or other basic estimates (e.g. regression coefficient) AND variation (e.g. standard deviation) or associated estimates of uncertainty (e.g. confidence intervals)
☒	☐	For null hypothesis testing, the test statistic (e.g. *F*, *t*, *r*) with confidence intervals, effect sizes, degrees of freedom and *P* value noted<br>*Give P values as exact values whenever suitable.*
☒	☐	For Bayesian analysis, information on the choice of priors and Markov chain Monte Carlo settings
☒	☐	For hierarchical and complex designs, identification of the appropriate level for tests and full reporting of outcomes
☒	☐	Estimates of effect sizes (e.g. Cohen's *d*, Pearson's *r*), indicating how they were calculated

Our web collection on statistics for biologists contains articles on many of the points above.

Software and code

Policy information about availability of computer code

Data collection	EPU (ThermoFisher)
Data analysis	cryoSPARC v4.0.0, Relion v3.1.3, Phenix 1.21.1-5286-000, Coot 0.9.8.92, ISOLDE 1.6.0, AlphaFold2, UCSF Chimera 1.17.1/ChimeraX 1.7.1, PyMOL 3.0.2, GeneSys software (Syngene, v1.8.5.0), GraphPad Prism 6.0

For manuscripts utilizing custom algorithms or software that are central to the research but not yet described in published literature, software must be made available to editors and reviewers. We strongly encourage code deposition in a community repository (e.g. GitHub). See the Nature Portfolio guidelines for submitting code & software for further information.

Data

Policy information about availability of data

All manuscripts must include a data availability statement. This statement should provide the following information, where applicable:

- Accession codes, unique identifiers, or web links for publicly available datasets
- A description of any restrictions on data availability
- For clinical datasets or third party data, please ensure that the statement adheres to our policy

Coordinates of the BcsA-BcsB6_BcsG3 complex have been deposited at the PDB alongside the cryo-EM map with accessing codes 9B8V and EMD-44359. The coordinates for the BcsB-BcsC complex, BcsC in the absence and the presence of cellotetroase, and the BcsZ tetramer have been deposited at the Protein Data Bank with accessing codes 9B8I and EMD-44346, 9B8A and EMD-44336 and 9B8H and EMD-44345, and 9B87 and EMD-44334, respectively.

Research involving human participants, their data, or biological material

Policy information about studies with human participants or human data. See also policy information about sex, gender (identity/presentation), and sexual orientation and race, ethnicity and racism.

Reporting on sex and gender	Not Applicable
Reporting on race, ethnicity, or other socially relevant groupings	Not Applicable
Population characteristics	Not Applicable
Recruitment	Not Applicable
Ethics oversight	Not Applicable

Note that full information on the approval of the study protocol must also be provided in the manuscript.

Field-specific reporting

Please select the one below that is the best fit for your research. If you are not sure, read the appropriate sections before making your selection.

[x] Life sciences [ ] Behavioural & social sciences [ ] Ecological, evolutionary & environmental sciences

For a reference copy of the document with all sections, see nature.com/documents/nr-reporting-summary-flat.pdf

Life sciences study design

All studies must disclose on these points even when the disclosure is negative.

Sample size	Cryo-EM data collection was performed in order to attain sufficient number of particles for authentic 3D classification and generating high quality EM maps. Each activity assay was performed in technical triplicates.
Data exclusions	No data is excluded from analysis except in the regular cryo-EM data processing which involves discarding of poor quality images and particles.
Replication	Enzymatic assays were performed in triplicates, Cellulase and Congo-red binding and fluorescence plate assays were performed in technical triplicates with at-least two biological replicates. PACE analysis was done two times.
Randomization	Not Applicable
Blinding	Not Applicable

Reporting for specific materials, systems and methods

We require information from authors about some types of materials, experimental systems and methods used in many studies. Here, indicate whether each material, system or method listed is relevant to your study. If you are not sure if a list item applies to your research, read the appropriate section before selecting a response.

Materials & experimental systems

n/a	Involved in the study	
[ ]	[x]	Antibodies
[x]	[ ]	Eukaryotic cell lines
[x]	[ ]	Palaeontology and archaeology
[x]	[ ]	Animals and other organisms
[x]	[ ]	Clinical data
[x]	[ ]	Dual use research of concern
[x]	[ ]	Plants

Methods

n/a	Involved in the study	
[x]	[ ]	ChIP-seq
[x]	[ ]	Flow cytometry
[x]	[ ]	MRI-based neuroimaging

Antibodies

Antibodies used	Commercially available antibodies are used for detection of recombinant proteins by Western Blotting: Monoclonal mouse penta-His Antibody, BSA free from Qiagen (Cat# 34660; dilution 1:2000), Monoclonal Anti-Flag M2 antibody produced in mouse from Sigma (Cat# F3165; dilution 1:10000), Anti-Mouse IgG (H&L) (Goat) Antibody DyLight 800 Conjugated (Cat# 610-145-002; dilution 1:5000).

Validation	Provided by manufacturers

Plants

Seed stocks	Not Applicable
Novel plant genotypes	Not Applicable
Authentication	Not Applicable
